# Serotonin-1A receptor C-1019G polymorphism affects brain functional networks

**DOI:** 10.1038/s41598-017-12913-3

**Published:** 2017-10-02

**Authors:** Haixia Zheng, Keiichi Onoda, Yasuko Wada, Shingo Mitaki, Toru Nabika, Shuhei Yamaguchi

**Affiliations:** 1 Department of Neurology, Faculty of Medicine, Shimane University, 89-1 Enya-cho, Izumo, Shimane 693-8501 Japan; 2 Department of Functional Pathology, Faculty of Medicine, Shimane University, Izumo, Japan

## Abstract

The serotonin-1A (5-HT1A) receptor is strongly implicated in major depression and other affective disorders due to its negative regulation of serotonin neurone firing rates. Behavioural and clinical studies have repeatedly reported that the −1019G allele carries a high susceptibility for affective disorders. However, the underlying pathophysiology remains unknown. Here, we employed a genetic neuroimaging strategy in 99 healthy human subjects to explore the effect of serotonin-1A receptor polymorphism on brain resting-state functional connectivity (FC). We used functional magnetic resonance imaging, along with a seed-based approach, to identify three main brain networks: the default mode network (DMN), the salience network (SN) and the central executive network. We observed a significant decrease in the FC of the DMN within the dorsolateral and ventromedial prefrontal cortices in G-carriers. Furthermore, compared with the C-homozygote group, we observed decreased FC of the SN within the ventromedial prefrontal cortex and subgenual anterior cingulate cortex in the G-carrier group. Our results indicate that 5-HT1A receptor genetic polymorphism modulates the activity of resting-state FC within brain networks including the DMN and SN. These genotype-related alterations in brain networks and FC may provide novel insights into the neural mechanism underlying the predisposition for affective disorders in G allele carriers.

## Introduction

5-Hydroxytryptamine (5-HT), more commonly known as serotonin, is the most widely distributed neurotransmitter in the brain, and dysfunctions of the serotonergic system are thought to be important factors for major depressive disorder and other forms of affective disorders^1–3^. Recent evidence suggests that the higher expression of the 5-HT1A presynaptic autoreceptor in raphe nuclei reduces the firing rate of serotonergic neurons and negatively regulates 5-HT release^4^. *In vivo*, human positron emission tomography (PET) studies have demonstrated that the expression of autoreceptors is modulated by a 5-HT1A receptor gene C(−1019)G polymorphism, and, specifically, that the binding potential is increased with increasing frequency of the G allele^5,6^. This is associated with increased negative feedback and, consequently, decreased serotonin signalling. In accordance with these findings, clinical studies have also reported that the −1019G allele is associated with fear expression^7^, trait anxiety^8^, schizophrenia^9^, major depression and suicide^10^. Therefore, genetic variations in the 5-HT1A receptor appear to play a critical role in affective disorders and need further investigation.

Resting-state functional Magnetic Resonance Imaging (fMRI), a noninvasive, nonradiation means for mapping the functional connectivity (FC) of the human brain, has become a promising avenue to investigate the neurophysiological basis of human brain functions. The well-known large scale networks of default mode network (DMN), salience network (SN) and central executive network (CEN) have been suggested to play a fundamental role in supporting emotional and/or cognitive functions^11^ and are strongly implicated in an array of affective disorders such as depression^12^ and schizophrenia^13^. Previous neuroimaging studies using fMRI spontaneous fluctuations in blood oxygen level-dependent (BOLD) signals have provided valuable insights into the effects of 5-HT1A receptor on FC. In an animal study, an increase of FC in the cortex, hippocampus, amygdala and dorsomedial thalamus was observed after giving 5-HT1A receptor agonist, 8-OH-DPAT^14^. These brain areas are known to express high levels of the 5-HT1A receptor^15,16^ and are involved in the frontal cortex-basal ganglia circuits that play critical roles in affective disorders^17^. Human studies, using combined PET and resting-state fMRI, have also confirmed that raphe nuclei have significant positive FC within brain regions involved in cognitive and emotion processing and that the 5-HT1A receptor binding potential appears to predict the activity of DMN^18,19^. It has been demonstrated that 5-HT1A receptor modulates brain FC. Yet, it is unknown whether this modulation is affected by the 5-HT1A receptor genetic variant.

Genetic studies have confirmed that the −1019G allele abolishes the transcription factor human nuclear deformed epidermal autoregulatory factor-1 (DEAF-1) and partially impairs Hairy/Enhancer-of-split-5 (Hes5), resulting in an increase in 5-HT1A receptor expression at the presynapse of serotonin-containing neurons of the raphe nuclei, whereas it decreases postsynaptic 5-HT1A receptor expression in non-serotonin-containing neurons of the forebrain (hippocampus, cortex)^5,10,20,21^. Furthermore, during emotional processing tasks, the 5-HT1A C(−1019)G polymorphism has been shown to impact the activity of forebrain targets, such as the amygdala^8^, insula and anterior cingulate^7^. Interestingly, as in affective function, the G allele in healthy participants is also associated with a decrease in cognitive ability such as in error detection and attentional processing^22,23^. Taken together, we therefore hypothesised that the C(−1019)G 5-HT1A receptor polymorphism is a potential neurochemical modulator for FC in human brain networks that associate with emotional and/or cognitive processing.

To summarise, in the current study, we aim to fill this gap in the known effects of 5-HT1A C(−1019)G variant on resting-state brain networks. We used resting-state fMRI to investigate how the polymorphism of the 5-HT1A receptor affects brain functional networks and whether it is correlated with emotional and cognitive function in healthy adults. We examined three main large-scale networks known to underlie a broad range of human emotional-cognitive operations: the DMN, which is a network important in self-referential activities^24^; the SN, which is critical for emotional awareness; and the CEN, a network associated with emotional regulation and top-down cognitive control of attention^24,25^.

## Materials and Methods

### Participants

The current study included a total number of 120 Japanese participants (77 males, 43 females) who were voluntarily participating in the brain check-up system at the Shimane University Institute of Health Science. The mean age of the participants was 56.3 ± 14 (SD) years. All participants reported free of neurological and psychiatric disorders and brain injury. They all underwent resting-state fMRI, blood test and neuropsychological examinations. Formal physical and neurological examinations were performed by an experienced neurologist based on a detailed medical interview, structural MRI and blood test. We excluded 21 participants who showed abnormalities in structural MRI: 8 participants who exhibited apparent cerebral microbleeds, 7 with silent brain infarction and 6 with pathological subcortical white matter lesions. Finally, 99 healthy participants (65 males, 34 females, mean age 53.9 ± 13.1 (SD) years, ranging from 34 to 87 years old) were included in the statistical analysis. Permission to conduct the study was approved by the Shimane University Ethics Committee and all methods were performed in accordance with the relevant guidelines. All participants gave informed written consent. The health check-up report was given as feedback to all participants. There was no monetary compensation for their participation in this study.

### Neuropsychological Assessment

All participants were assessed using neuropsychological test batteries including the Mini-Mental State Examination (MMSE)^26^, the Frontal Assessment Battery (FAB)^27^ to measure cognitive function, and the Self-rating Depression Scale (SDS)^28^ and the Apathy Scale (AS)^29,30^ to measure emotional function.

### Genotyping for the 5-HT1A Receptor

Genotypic analysis for 5-HT1A receptor (1019C/G) polymorphisms was carried out using the TaqMan SNP Genotyping Assay system (Assay ID: C_11904666_10; Applied Biosystems, Foster, CA, USA) as previously described in other studies by our group^31^. Briefly, fasting venous blood samples were obtained from all study participants. Genomic DNA was extracted from peripheral blood leukocytes using a standard phenol/chloroform method. Genotyping of the C-1019G polymorphism in the 5-HT1A receptor was amplified using polymerase chain reaction (PCR). Briefly, 10 ng of DNA was amplified in a total volume of 5 µL reaction in a 384-well plate, containing the SNP-specific MGB process for the 5-HT1AR SNP (rs6295) (Applied Biosystems) and 2.5 µL of TaqMan genotyping master mix (Applied Biosystems). The PCR was then performed using thermal cycling conditions as follows: after the first step at 50 °C for 2 min, 95 °C for 10 min, steps of 92 °C for 15 s and 60 °C for 1 min for 40 cycles. After PCR amplification, an allelic discrimination plate read was performed using an Applied Biosystems Real-Time PCR System. Finally, allelic discrimination analysis was carried out using ABI Prism 7900HT Sequence Detection System Software (Applied Biosystems).

### Image Acquisition

All functional and structural imaging data were acquired using a Siemens 1.5T MRI scanner (Erlangen, Germany). Participants were instructed to remain still with eyes closed and to try to avoid engaging in any thinking but to not fall asleep during the resting-state fMRI scan. Twenty-seven axial slices parallel to the plane connecting the anterior and posterior commissures were measured using a T2*-weighted gradient echo spiral pulse sequence (repetition time = 2000 ms, echo time = 28 ms, flip angle = 90°, scan order = interleave, matrix size = 64 × 64, field of view = 256 × 256 mm^2^, in-plane resolution = 4 × 4 mm^2^, slice thickness = 4.5 mm, gap = 0 mm, duration = 5 min). After 5-min rs-fMRI scans, T1-weighted images of the entire brain were measured (192 sagittal slices, repetition time = 1740 ms, echo time = 3.22 ms, inversion time = 1100 ms, flip angle = 15°, matrix size = 256 × 256, field of view = 256 × 256 mm^2^, isotropic spatial resolution = 1 mm).

### Functional Imaging Processing

Image preprocessing was carried out with SPM12 (Wellcome Trust Centre for Neuroimaging, London, UK, http://www.fil.ion.ucl.ac.uk/spm/software/spm12/) running under Matlab R2016a (The MathWorks, Inc., Natick MA, USA). The following processing was applied: first, slice-timing correction and head motion correction were conducted. Then the corresponding anatomical (T1- weighted) image was co-registered to the first EPI image. Afterward, normalisation to SPM12’s EPI anatomical volume within a standard stereotactic space (the T1-weighted template from the Montreal Neurological Institute, MNI) was performed using the DARTEL method, with resampling to 3 × 3 × 3 mm.

Seed-based correlation analysis was performed to establish the DMN, SN, and CEN due to its straightforward interpretability and high reliability^32,33^. Seed-to-voxel FC analyses were performed using the CONN FC toolbox v16^34^ (http://www.nitrc.org/projects/conn). Head motion was defined as a first level covariate, and white matter and CSF BOLD signals were defined and addressed in the default setting. A component-based noise-correction ‘CompCor’ strategy utilised in this toolbox^35^ was used to control physiological and movement confounds. Band-pass filtering was set to a frequency window from 0.007 to 0.080 Hz.

To avoid seed selection bias, we followed Andreas’s approach for computed FC networks in several steps^19^. In detail, two predefined seed masks (cubic volume of 3 mm × 3 mm × 3 mm with MNI coordinates, voxels = 216 mm^3^) were created using WFU_PickAtlas toolbox^36,37^ (http://fmri.wfubmc.edu/software/pickatlas) and were based on previous literature as seeds to create connectivity maps of the default mode network^38^. One was defined within the posterior cingulate cortex (x/y/z = 0/−52/30 mm), and the other one was created from a seed within the medial prefrontal cortex (x/y/z = 0/50/22 mm). Two binary maps of the DMN were obtained by applying one sample t-tests across subjects with a significance threshold of *p* < 0.001 and family-wise error corrected (FWE-corrected) at the voxel level for multiple comparisons. The final DMN seed region was then calculated as the intersection of these two binary DMN masks. Here, the obtained final DMN seed mask contained a conjunction of DMN core regions such as posterior cingulate, medial prefrontal and lateral parietal cortex rather than a particular region. After that, the BOLD signal from the final DMN seed region was averaged, and this was taken as a seed time course. The seed-to-voxel FC followed by z values maps were then created for each participant. Bivariate regression analyses were used to determine the linear association of the BOLD time series between the seed and each voxel. Hence, this approach enabled an interpretation of the resulting z-values not only in terms of connectivity between two regions but also as the functional connectivity between each voxel and the network obtained from the current iterative seed selection strategy. Similarly, the CEN and SN were defined using the same strategy. The seed mask of the CEN was created from the left and right dorsolateral prefrontal cortex (L:x/y/z = −42/34/20 mm; R:x/y/z = 44/36/20 mm)^13,39,40^, whereas the seed mask of the SN used the left and right frontal insular cortex (L:x/y/z = −34/12/−2.5 mm; R:x/y/z = 34/12/−2.5 mm)^41^.

### Statistical Analysis

To investigate the effect of 5-HT1A receptor (1019C/G) polymorphisms on resting-state FC, second level analyses of two sample t-tests were performed using SPM12. Due to the strong correlation between age and resting-state network FC^42^, age was taken as a covariate. Because the G-homozygote has been reported to be involved in the decline of cognitive function among women with premenstrual dysphoric disorder^43^, we took sex as another covariate. SPM12 default threshold of *p* < 0.001 was set for bidirectional explorations of connectivity. Results of exploratory analyses were considered significant if clusters survived FWE correction at *p* < 0.05.

We ran correlation analyses separately for each group to explore the relationship between group differences obtained from seed-based analysis and neuropsychological function, removing the effects of age and sex using partial correlations.

### Data availability

The data that support the findings of this study are available from the corresponding author on reasonable request.

## Results

### Genotyping

Genotyping was performed in all 120 samples. In order to control genotyping error, control samples with known genotypes were added to each 384-well plate. Genotype distribution of the C(−1019)G polymorphism was checked by the Chi-square test and was consistent with Hardy-Weinberg Equilibrium (*X* ^2^ = 2.62, *p* = 0.11). The minor allele frequency (G allele) was 0.267 (CC:68, CG:40, GG:12) which was consistent with the general Japanese population (0.247)^9^. Since 21 participants showed abnormalities in structural MRI, they were excluded from the following analyses. As a result, 99 samples without abnormal structural MRI findings (CC:59, CG:28, GG:12) were used in the analyses of the effect of 5-HT1A receptor polymorphism on brain FC. Due to the limited sample size and previous reports supporting this approach^44^, we decided a priori to dichotomise subjects into carriers of the putative risk allele as G-carriers and C-homozygotes. There were no significant differences in age, gender and neuropsychological scores between G-carrier and C-homozygote groups (Table 1).

**Table 1:** Genotyping results for the 5-HT1A receptor, neuropsychology score and demographic data.

Variable	CC	GG/CG	*p*
N	59	40	—
Age	54.4(12.3)	53.1(14.2)	0.62^b^
Male:Female	38:21	27:13	0.75^a^
MMSE	28.8 (1.8)	28.7 (1.5)	0.80^b^
FAB	17.0 (1.1)	17.2 (1.4)	0.54^b^
SDS	35.4 (7.6)	35.4 (7.1)	0.98^b^
AS	11.4 (6.1)	11.9 (6.2)	0.68^b^

Data are presented as mean ± SD. Abbreviations: CC,Chomozygotes; GG/CG, G-carriers; MMSE, Mini-Mental State Examination; FAB, Frontal Assessment Battery; SDS, Self-rating Depression Scale; AS, Apathy Scale. a, calculated by Chi-square test; b, calculated by two sample *t*-test.

### Resting-state FC Networks

**Figure 1:**
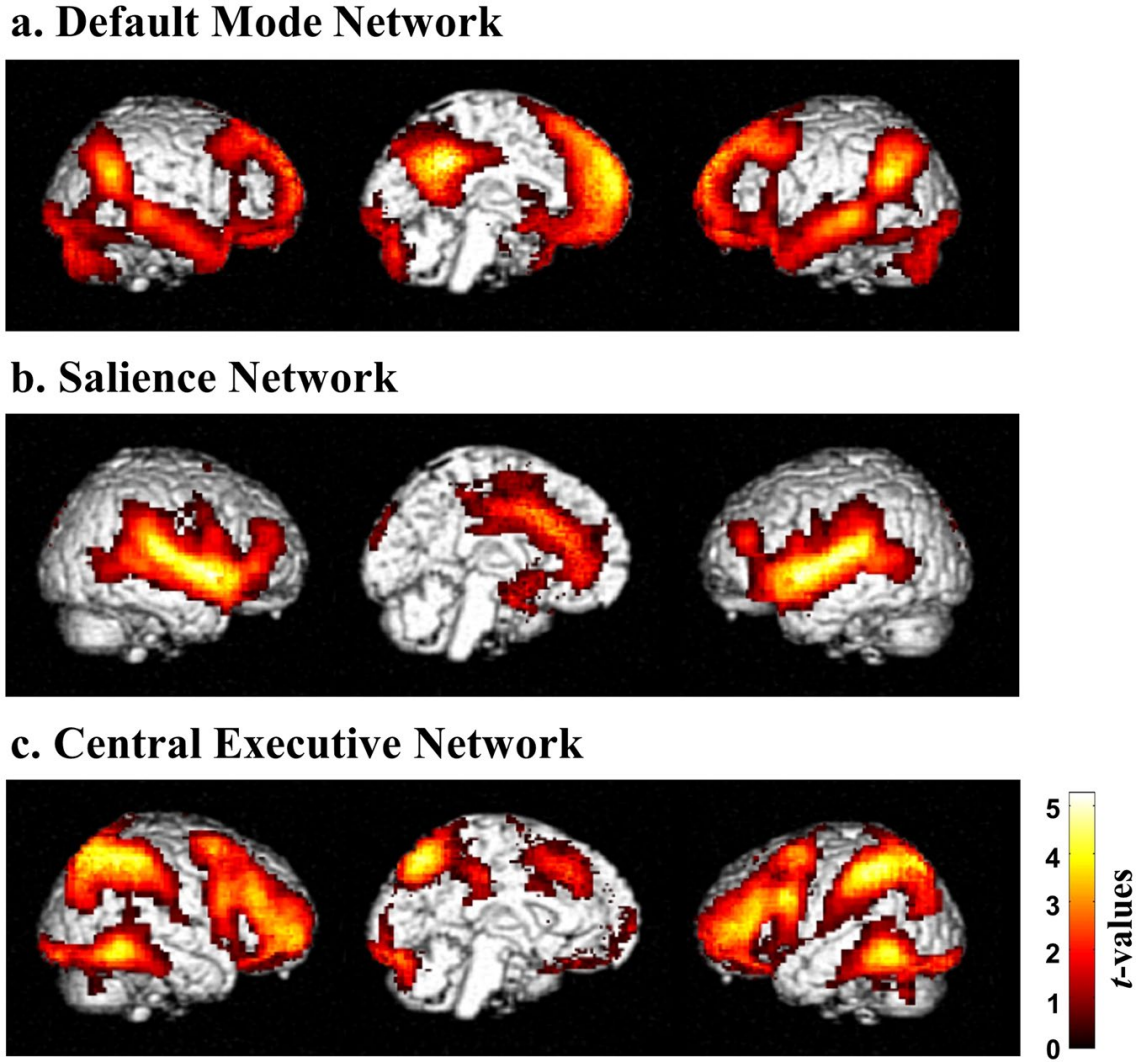
Average functional connectivity maps across 99 participants computed for BOLD time series using seed-based analysis (cluster-level family-wise error corrected, *p* < 0.001). These connectivity maps are independent representations of (**a**) the default mode network, (**b**) the salience network and (**c**) the central executive network. The functional connectivity of the networks is shown in the left lateral, midline and right lateral views from left to right.

The DMN, SN, and CEN were assessed using seed-based analysis. The core region of DMN, SN, CEN were consistent with prior study^13^. The FC analysis showed substantial involvement of the posterior cingulate, medial prefrontal and lateral parietal cortices in the DMN (Fig. 1a). The SN consisted of the dorsal anterior cingulate cortex and bilateral insula (Fig. 1b), whereas the CEN involved the dorsolateral prefrontal cortex and other regions such as the bilateral superior parietal and inferior temporal gyri (Fig. 1c).

### Comparison of Resting-state FC Between G-homozygote and C-carrier Groups

**Figure 2:**
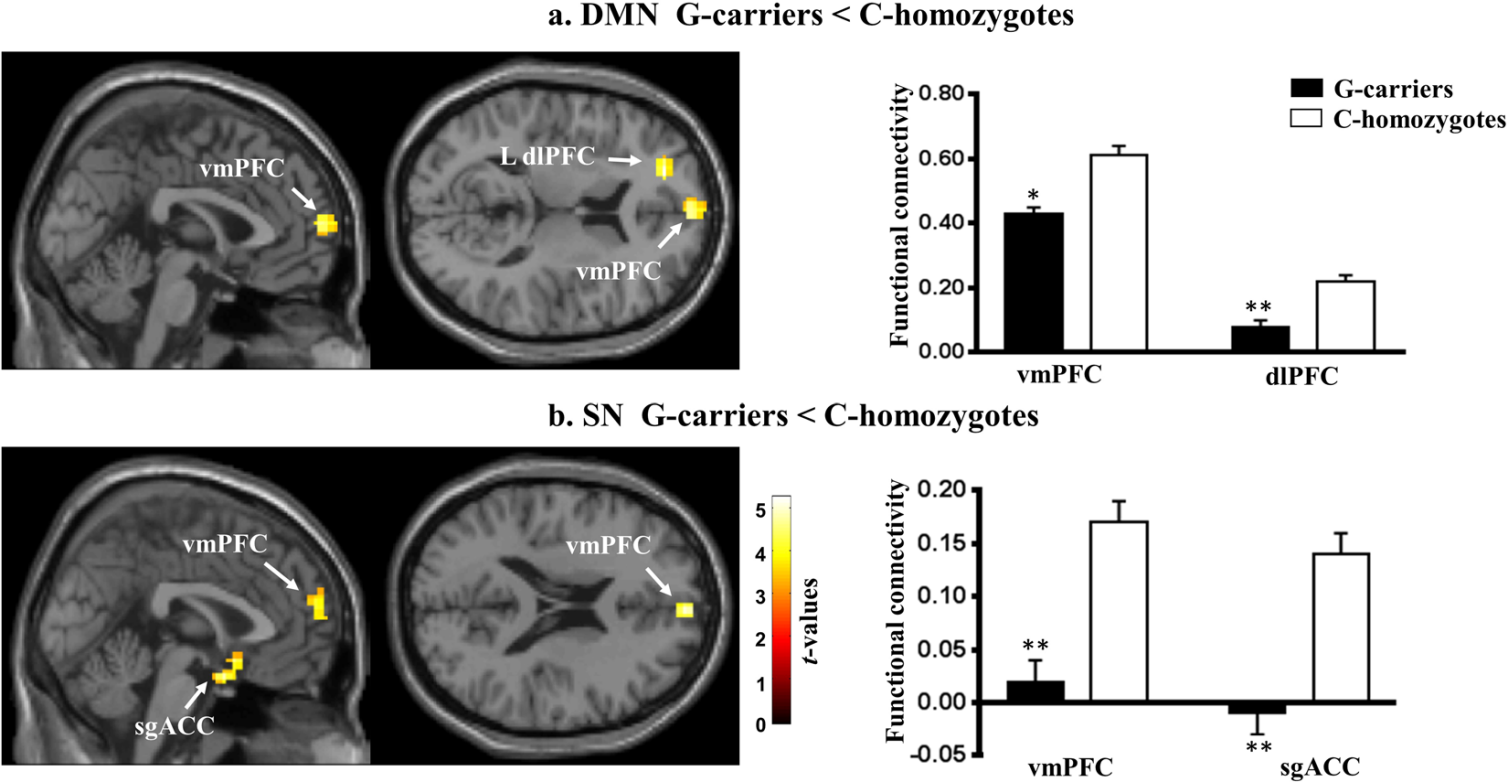
Genotype group differences in functional connectivity (FC) in the default mode network (DMN) and salience network (SN). (**a**) Spatial maps illustrating that the regions of the left dorsolateral prefrontal cortex (dlPFC) and ventromedial prefrontal cortex (vmPFC) shown functional hypoactivation within the DMN in the G-carrier group compared to the C-homozygote group. Bar graphs show the mean FC for each group in the vmPFC and left-dlPFC, demonstrating a significant decrease in FC activity in the G-carrier group. (**b**) Spatial maps illustrating that the regions of the vmPFC and subgenual anterior cingulate cortex (sgACC) shown functional hypoactivation within the SN in the G-carrier group. Bar graphs show the mean FC for each group in the vmPFC and sgACC, demonstrating a significant decrease in FC activity in the G-carrier group. (Asterisks indicate a significant difference between groups, cluster-level family-wise error corrected, *p < 0.05, **p < 0.01. Error bars represent standard errors of the mean).

We compared resting-state FC between G-carrier and C-homozygote groups. G-carriers showed decreased cortical FC of the left dorsolateral prefrontal cortex (L-dlPFC, cluster-level FWE-corrected, *p* = 0.004, Fig. 2a) and ventromedial prefrontal cortex (vmPFC, cluster-level FWE-corrected, *p* = 0.016, Fig. 2a) for the DMN. In the SN, there was a significant decrease in FC at the vmPFC (cluster-level FWE-corrected, *p* = 0.001, Fig. 2b) and subgenual anterior cingulate cortex (sgACC, cluster-level FWE-corrected, *p* = 0.002, Fig. 2b) was also observed in the G-carrier group. No areas where cortical FC was significantly increased in the G-carrier group relative to the C-homozygote group were observed in the DMN or SN. There were no significant differences in FC within the CEN between the two groups. The anatomical locations of the voxels, which showed significantly different FC, are listed in Table 2.

**Table 2:** Resting-state network alterations in the 5-HT1A receptor C−1019G polymorphism.

Network	Contrast	Region	MNI coordinate			Peak *t*-value	Cluster size (voxels)*
			X	Y	Z		
DMN	G < CC	dlPFC(L)	−20	48	14	5.12	203
		vmPFC	0	62	12	4.51	157
	G > CC	None	—	—	—	—	—
SN	G < CC	sgACC	−2	12	−22	5.21	227
		vmPFC	4	60	20	4.95	260
	G > CC	None	—	—	—	—	—
CEN	G < CC	None	—	—	—	—	—
	G > CC	None	—	—	—	—	—

Voxel size = 3*3*3 mm. Abbreviations: G = G-carriers; CC = C-homozygotes; dlPFC = Dorsolateral Prefrontal Cortex; vmPFC = Ventromedial Prefrontal Cortex; sgACC = Subgenual Anterior Cingulate Cortex; L = Left.

### Correlation Between Resting-state FC and Neuropsychology

Correlation analyses between neuropsychological scores and resting-state FC were performed with age and sex as covariates. There were no significant correlations between resting-state network connectivity and neuropsychological scores.

## Discussion

The goal of the present study was to investigate whether 5-HT1A receptor polymorphism impacts FC of the large-scale networks for DMN, SN, and CEN. We report two main findings: First, G-carriers showed hypoconnectivity of DMN seeds within the dlPFC and vmPFC compared to the C-homozygote group. Second, G-carriers showed a substantial decrease in the FC of SN seeds within the vmPFC and sgACC.

Our results confirmed that the 5-HT1A receptor modulates FC in the DMN^19^ and extended the results of previous neuroimaging studies by showing a strong effect of this single nucleotide polymorphism on the DMN and SN. The BOLD signal, which we used to calculate the FC and identify the brain networks, has been reported to be locally driven by glutamatergic signalling and globally by monoamine neurotransmitter signalling, including serotonin^45^. Furthermore, serotonin neurons in the raphe nuclei that send projections to the forebrain mediate glutamatergic and GABAergic signalling^46,47^.In addition, genetic studies have demonstrated that -1019G allele altered the transcriptional regulation and is associated with increased 5-HT1A autoreceptor binding and decreased postsynaptic 5-HT1A receptor in the forebrain, leading to a decrease of serotonin signalling^10,20,21^. Collectively, these findings suggest it is possible that −1019G allele mediates decreased serotonin signalling, consequently decreased BOLD signal in fMRI. Consistent with these findings, recent evidence from PET-fMRI approaches has revealed that 5-HT1A receptor binding sites overlap the core brain regions of the DMN and SN. It has also been elucidated that the density of the 5-HT1A receptor regulates the connectivity of front-limbic circuits, both structurally and functionally, and is strongly associated with emotional regulation^5,18,19,48–50^. Hence, in line with these previous reports, our finding of decreased FC of the prefrontal cortex area and sgACC within the DMN and SN in the G-carrier group suggests a central role for 5-HT1A receptor genetic polymorphism in the modulation of front-limbic circuit function. In addition, it provides a better understanding of serotonin-related inter-individual variability in human brain large-scale networks activity.

Previous studies have highlighted that the vmPFC exerts control over neural activity in the dorsal raphe nucleus, a serotonergic nucleus, which affects the level of 5-HT1A and certain behaviours^51^. Task-based neuroimaging studies have reported that the vmPFC is related to affect processing when one is facing uncertainty, viewing happy and fearful faces or experiencing anticipatory anxiety or negative affects^52,53^. People with vmPFC lesions exhibit significant alterations of ambiguous cue-related activity of the insula, which is the hub region of the SN^54^. Interestingly, we observed a significant decrease of FC in the similar area of the vmPFC within both DMN and SN in the G-carrier group, leading us to speculate that −1019G allele is involved in affective processing through affecting resting-state network’s function. Furthermore, sgACC activity has been reported to be positively correlated with depressive symptoms^55^, and the FC between this area and the amygdala is positively correlated with harm avoidance score^56^. From a clinical perspective, the FC in the dorsal medial part of the PFC, a hub of the DMN, has been reported as a ‘hot wiring’ in major depressive patients when compared with healthy controls^12^. Conversely, other resting-state fMRI studies revealed that medial PFC exhibited decreased FC within large-scale networks such as DMN and SN in patients with depression^57,58^. Taken together, existing evidence has the capacity to confirm that the involvement of the abnormal FC in the vmPFC region is associated with the pathology of affective disorders. However, there is no consistent pattern of abnormalities in large-scale functional brain networks. Our results provide an initial evidence to associate the 5-HT1A receptor C(−1019)G polymorphism with the hypoconnectivity of vmPFC within DMN and SN. This could contribute to the understanding of neural circuitries underlying the influence of −1019G allele on complex FC alteration implicated with affective disorders.

The CEN involving the dlPFC and parietal regions is critical in cognitive control processes^59^. The abnormal FC of CEN and/or dlPFC region has been linked to several psychiatric disorders such as major depression, bipolar and schizophrenia^57,60^. In addition, a significant decrease of 5-HT1A mRNA in subjects with major depression in dlPFC region has been reported in a postmortem brain study^61^. We expected to find associations between 5-HT1A receptor gene variants with FC in DMN, SN, and CEN. However, we did not observe any differences in the FC of the CEN but only within the DMN and SN between G-carriers and C-homozygotes. Consistent with our result, a task-based fMRI study which investigated the association of 5-HT1A C(−1019)G polymorphism with brain region activity in patients with panic disorder also observed a decreased activity of vmPFC but not dlPFC in −1019G allele carriers^62^. Thus, the polymorphism of 5-HT1A receptor might not directly impact higher cognitive processing in healthy human brains.

In order to control the effect of the structural abnormalities on FC, we had to exclude 21 participants with abnormalities in structural MRI from statistical analyses. This manipulation resulted in a significant distortion of genotype frequencies from the Hardy-Weinberg Equilibrium (HWE, *X* ^2^ = 7.2, *p* = 0.007). However, as shown in the Results section the genotype frequencies of the total 120 samples was consistent with the HWE, which indicated that the genotyping procedure was adequately performed and the samples were not biased. In fact, all of the 21 participants had the minor C allele (9 CC and 12 CG), which might suggest an association between the C allele and the risk of silent brain lesions. Further investigation on the causal relationship between this SNP and silent brain lesions is warranted.

No correlation between FC and neuropsychological scores was found. To our knowledge, the vast majority of the previous studies that have linked G-homozygotes with affective disorders took place in a clinical setting. Our participants are healthy. Therefore, replication of 5-HT1A receptor polymorphism studies in clinical settings are necessary to confirm our findings and interpretations. The average age of our participants was 54, ranging from 34 to 87 years old. Thus, our results should be interpreted with caution since it has been reported that the number of available 5-HT1A binding sites declines with age^63^. We were also limited in our data acquisition process. The duration of our resting-state scan was 5 min. However, we cross-checked our results with another study to ensure that we had successfully identified the proper brain FC networks^13^.

In summary, we found that 5-HT1A receptor polymorphism modulates brain FC of the DMN and SN, with these areas engaging in emotional regulation, and that G-carriers have decreased FC activity in regions related to emotional processing. Our findings suggest that 5-HT1A receptor polymorphism may impact the brain’s emotional regulation process, thus offering new evidence and strategic insight into the underlying mechanisms of 5-HT1A receptor genetic susceptibility for affective disorders.
